# Continuous Flow Synthesis of Propofol

**DOI:** 10.3390/molecules26237183

**Published:** 2021-11-26

**Authors:** Romain Mougeot, Philippe Jubault, Julien Legros, Thomas Poisson

**Affiliations:** 1 CNRS, INSA Rouen, UNIROUEN, Normandie University, COBRA, 76000 Rouen, France; mougeot.romain@gmail.com; 2 Institut Universitaire de France, 1 Rue Descartes, 75231 Paris, France

**Keywords:** active pharmaceutical ingredient, anesthetics, Friedel–Crafts, decarboxylation

## Abstract

Herein, we report a continuous flow process for the synthesis of 2,6-diisopropylphenol—also known as Propofol—a short-acting intravenous anesthesia, widely used in intensive care medicine to provide sedation and hypnosis. The synthesis is based on a two-step procedure: a double Friedel–Crafts alkylation followed by a decarboxylation step, both under continuous flow.

## 1. Introduction

**Scheme 1:**
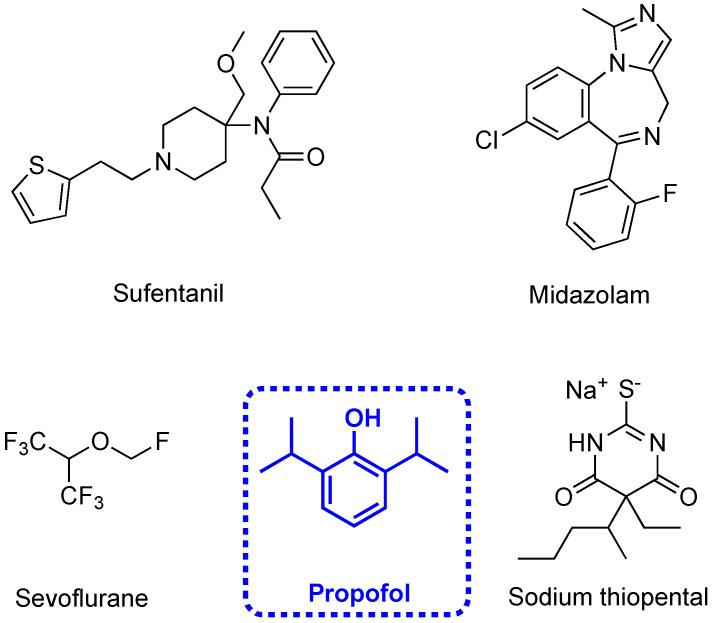
Example of different anesthetics.

Propofol (2,6-diisopropylphenol) is a potent intravenous hypnotic agent, which is widely used for the induction and maintenance of anesthesia and for sedation in the intensive care units [1]. Propofol is chemically distinct from others intravenous sedative hypnotic such as opioids, barbiturics, halogenated liquids, or benzodiazepines (Scheme 1) [2]. Propofol is characterized by a rapid onset due to its high lipophilicity, a short duration of action and a low toxicity due to its rapid hepatic and extra-hepatic metabolization into various salts, which are excreted in urine. Moreover, it provides satisfactory sedation and fast recovery time. Hence, Propofol has been recognized as an essential medicine by the World Health Organization since April 2013 [3].

Importantly, within the context of the COVID-19 pandemic, Propofol is extensively used to avoid cardio-pulmonary injuries for patients, who are mechanically ventilated by minimizing resistance to this mechanical ventilation [2]. At the early stage of this pandemic, a dramatic shortage of such strategic drugs was witnessed.

Although, most of the drugs are produced under batch conditions, continuous flow chemistry has emerged as a possible alternative for the production of active pharmaceutical ingredients (API) [4,5,6], especially since it offers a safer handling of chemicals minimizing the exposition of the operators and allows an easier scale-up [7,8,9,10,11,12,13]. Thus, following our ongoing research program dedicated toward the synthesis of API under continuous flow [14], we report our contribution for the first synthesis of the strategic Propofol under continuous flow.

## 2. Results

**Scheme 2:**
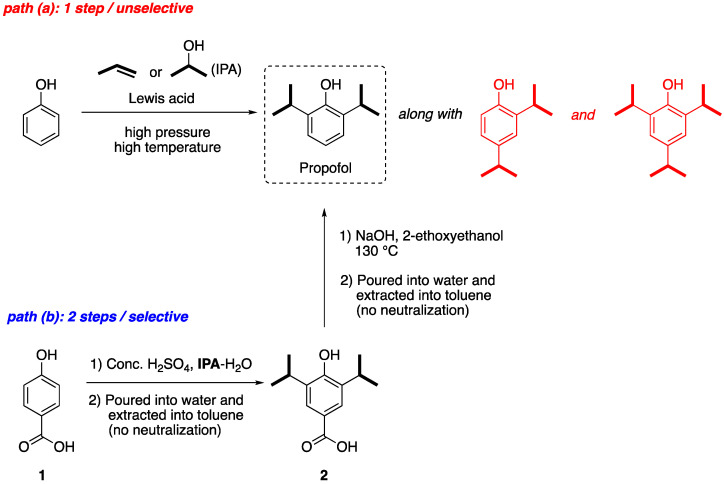
Path (**a**): Seminal synthesis of propofol. Path (**b**) Modified synthesis of Propofol according to Pramanik [23].

Seminal synthetic approaches to Propofol relied on Friedel–Crafts alkylation of phenol with propylene gas in the presence of a Lewis acid at high temperature (300 °C) and pressure (3000 bar) (Scheme 2, path a) [15,16,17,18,19,20,21,22]. Although isopropanol has been proposed to replace the gaseous propylene, these harsh conditions all led to the production of several impurities (2,4-diisopropyl and 2,4,6-triisopropyl phenol, along with the product (Scheme 2, path a)). These side products need to be removed from the final API (<0.05%) for medical use [23]. To address the formation of these undesired products in the synthesis of Propofol, another approach was designed using the 4-hydroxybenzoic acid (**1**) as the starting material to hamper the undesired alkylation at the *para* position. [23] Thus, the alkylation of **1** using isopropyl alcohol (IPA) and H_2_SO_4_ followed by a decarboxylation step under alkaline conditions (NaOH) at high temperature, afforded Propofol with a higher purity, matching with the API synthesis standard. However, this procedure suffers from acid–base neutralization at each step, resulting in exothermic quench, a serious drawback for an industrial implementation of the process. This issue was further tackled by Pramanik, who simplifies the isolation and purification steps by getting rid of the acid–base neutralization, using a toluene/water mixture (Scheme 2b) [23].

Inspired by this contribution toward the batch synthesis of Propofol, we started our investigations on a continuous flow process from 4-hydroxybenzoic acid (**1**), as starting material, and studied the Friedel–Crafts alkylation with IPA to introduce the two isopropyl substituents. At the outset of our study, we faced a solubility issue with the solvent mixture initially envisioned to introduce the substrate **1** (H_2_SO_4_/IPA/water) by a single inlet in a heated coil reactor. Hence, to avoid any precipitation in the tube reactor, which would jeopardize the development of the continuous flow synthesis of the target, we implemented a set-up composed of two inlets feeding a PFA coil reactor (ID = 1.6 mm) equipped with a T-shaped mixer: inlet A contained the substrate **1** in a homogeneous H_2_SO_4_/H_2_O mixture (9:1, [1] = 0.4 M) and inlet B was composed of IPA (6 equiv./**1**) in a H_2_SO_4_/H_2_O mixture as well (9:1). Importantly, the stock solutions were preheated at 35 °C to avoid any possible clogging before the injection within the reactor.

Then, the influence of the flow rate—and hence the mixing efficiency—was studied by fixing the residence time at *t*^R^ = 40 min and adjusting the reactor length accordingly (Table 1). As described in Table 1, a higher flow rate afforded product **2** with very good isolated yields (entries 1 to 4). An optimum total flow rate of Q_T_ = 2.5 mL·min^−1^ (Q_A_ = 1.25 mL·min^−1^ and Q_B_ = 1.25 mL·min^−1^, reactor volume V = 100 mL) provided **2** with 84% yield. Importantly, the reaction was scaled up to 200 mmol (27.6 g of **1**, reaction productivity of **2**: 55.95 g·h^−1^), without loss of efficiency and the pure **2** was delivered with a high yield (84%) after a short filtration over a pad of silica gel (entry 4) [24].

Having optimized the conditions for an efficient bis-alkylation of **1** into **2**, we turned our attention to the final decarboxylation step to access Propofol. This second step, initially performed in batch with a solution of NaOH, 2.3 M in 2-ethoxyethanol at 130 °C for 12 h (Scheme 2a), was reinvestigated to fit, here again, with the continuous flow constraints. Thus, we switched from an inorganic base to an organic one; popular triethylamine (TEA), Hünig’s base (DIPEA) and tetramethylene ethylene diamine (TMEDA) were evaluated in various solvents systems (Table 2).

To achieve the synthesis of Propofol, the flow system was composed of a single inlet, containing a premixed solution of **2** with the appropriate base, which was introduced in a tubular copper reactor (ID = 1.0 mm, V = 10 mL) for a better thermal transfer and a putative assistance in the decarboxylation event (150 °C, *t*^R^ = 3 h). The system was equipped with a back pressure regulator (BPR) set at 9.5 bars to reach high temperatures. Since flow systems offer the possibility to telescope reactions, our studies began with solvent mixtures including toluene, the solvent used to extract **2** from the final the previous step (vide supra). Among bases assessed, TMEDA proved to be efficient, leading to fairly decent NMR yields (entries 1 and 2) compared to DIPEA (entries 3 and 4): ca. 60% of expected Propofol product vs. >20%. Note that similar results to those of TMEDA were obtained with the inexpensive TEA (entries 5–7) and an optimum of 2 equivalents was required. In addition, reactions performed with TEA afforded cleaner reaction mixtures (according to the GC and NMR analyses). Hence, the solvent optimization was pursued with TEA: switching from toluene/2-ethoxyethanol mixture to DMF (entry 9) or pure 2-ethoxyethanol (entry 10) allowed the formation of Propofol in very high yield (86%). Further optimization by increasing the temperature to 200 °C using 2-butoxyethanol allowed to reach 69% yield within 30 min (Q = 334 µL·min^−1^) at this higher temperature (entry 11). Thus, whereas the yield of the reaction is not formally improved, this parameter shall not be considered on its own under flow conditions. The reaction time is also an important factor that determines the productivity. Therefore, a residence time of 0.5 h at 200 °C appears more appealing than 3 h at 150 °C (entry 10 vs. entry 11).

**Figure 1:**
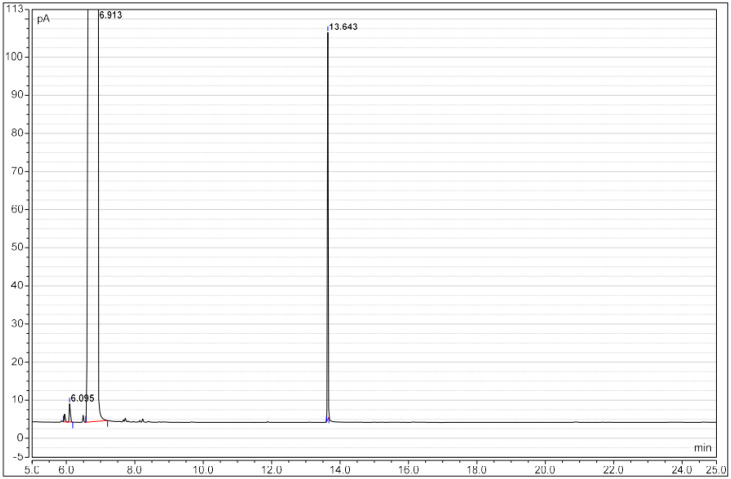
Gas chromatography of the crude mixture of the decarboxylation step (Table 2, entry 11). Propofol: *t*_R_ = 13.643 min, in the presence of the 2-butoxyethanol (*t*_R_ = 6.095 and 6.913 min).

The impurities profile for pharmaceutical compounds is fundamental for safety reasons, especially for intravenous drugs such as Propofol. Industrials processes allowing the Propofol have revealed the presence of numerous side products—which were detected by gas chromatography [25]—resulting from the synthesis and degradation, multiplying health’s risks and purification steps. Hence, to ascertain the purity level of the Propofol produced from our flow process, analyses by gas chromatography were performed. The collected data revealed that the crude mixture reached a very high level of purity, even without further purification (Figure 1). If required, a subsequent bulb-to-bulb distillation of the crude residue can even be performed.

## 3. Conclusions

In conclusion, we have reported herein the first continuous flow synthesis of the strategic Propofol anesthetic, recognized by the WHO within its essential list of medicines. This easy, practical, and safe two-step procedures afforded the Propofol in 70 min (vs. 16 h in batch) with an overall yield of 58% (84% yield for the Friedel–Crafts step and 69% yield for the decarboxylation step) close to the 64% described in batch (85% yield for the Friedel–Crafts step and 75% yield for the decarboxylation step). The final product is obtained with an excellent purity as shown by GC analysis. In addition, this practical continuous flow synthesis minimizes the exposure of the practitioner to concentrated sulfuric acid thanks to the use of a continuous flow reactor and a smoother quench of the acidic solutions (less exothermic). Finally, the decarboxylation reaction was smoothly addressed within 30 min thanks to an efficient and easy heat transfer. We do believe that this synthesis will pave the way for industrial endeavours to push this synthesis from a laboratory scale continuous flow synthesis to a highly productive continuous flow production manifold.

## 4. Materials and Methods

### 4.1. General Information

All materials were purchased from commercial suppliers. Unless specified otherwise, all reagents and solvents were used as supplied. ^1^H NMR spectra and ^13^C NMR spectra were recorded on a Bruker Advance III 300 (Bruker BioSpin Corporation, Billerica, MA, USA) at 300 MHz and 76 MHz, respectively (More details could be found on Supplementary Materials). Residual solvent peaks were used as the reference. Data for ^1^H are reported as follows: chemical shift (δ ppm), multiplicity (s = singlet; d = doublet; t = triplet; q = quartet; quint = quintet; sex = sextet; sept = septet; m = multiplet), coupling constant (Hz), and integration. Chemical shifts are reported in ppm relative to the signals corresponding to residual non-deuterated (CDCl_3_: δ = 7.26 ppm). Analyses were performed on GC-FID Thermoscientific trace 1310 equipped with Durabond DB-5MS (30 m, 0.250 mm Ø narrowbore, 0.25 µm film), the He vector gas flow rate of 1 mL·min^−1^ in split mode (50 mL·min^−1^) and the inlet injector temperature of 250 °C. As FID detector, the air flow rate is at 350 mL·min^−1^ with vector gas at 35 mL·min^−1^. Samples analyses were done after 2 min at 50 °C, at a temperature rate of 10 °C·min^−1^ for 20 min reaching 250 °C. All GC-sample were prepared using grade HPLC solvents from Fisher (LLC Hampton, NH, USA). Distillation were performed on a Büchi Kugelrohr. The flow reactor system is specified for each cases between the AFR^™^ Corning^®^ module pump device, the Vapourtec^®^ R4 heated system and R2S pump system.

### 4.2. Flow Synthesis of 3,5-diisopropyl-4-hydroxybenzoic Acid (**2**)

A stock solution of 4-hydroxybenzoic acid (**1**) (200 mmol, 27.6 g) in H_2_SO_4_/H_2_O (9:1, 445.5 mL/49.5 mL, 0.4 M) and a stock solution of isopropyl alcohol (6 eq., 1.2 mol, 71.5 g, 91.3 mL) in H_2_SO_4_/H_2_O (9:1, 363 mL/40.7 mL) were heated at 35 °C and pumped using the AFR^™^ Corning^®^ module pump at a flow rate of 1.25 mL·min^−1^ for each solution through a T-mixer heated at 60 °C and a 100 mL coil PFA reactor also heated at 60 °C. The output of the reactor was collected into a stirred solution of H_2_O/toluene (1:1) at room temperature. The setup was eluted by a solution of H_2_SO_4_/H_2_O (9:1). The layers were separated and the aqueous phase was extracted with toluene. The combined organics phases were washed with brine, dried with MgSO_4_, and concentrated under vacuum. Flash chromatography afforded the desired product **2** as a white solid (84%, 168 mmol, 37.3 g).

^1^H NMR (300 MHz, CDCl_3_) δ 7.87 (s, 2H), 3.17 (hept, *J* = 6.9 Hz, 2H), 1.31 (d, *J* = 6.9 Hz, 12H). ^13^C NMR (75 MHz, Chloroform-*d*) δ 172.64, 155.15, 133.73, 126.50, 121.47, 27.22, 22.60. Melting point: 139–142 °C. GC retention time: 19.02 min.

### 4.3. Flow Preparation of Propofol

To a stock solution of 3,5-diisopropyl-4-hydroxybenzoic acid **2** (0.5 mmol, 111 mg, 1 eq.) in 2-butoxyethanol (500 µL) was added triethylamine (3 mmol, 500 µL, 6 eq.). The mixture was stirred at room temperature and pumped by R2S Vapourtec^®^ pump device at a flow rate of 334 µL·min^−1^ through a 10 mL copper coil reactor at 200 °C and through a 1 mL copper coil reactor at room temperature. The setup was eluted by 2-butoxyethanol. The output of the reactor was collected into a stirred solution of brine/cyclohexane [26]. The organic layer was dried with MgSO_4_ and concentrated under vacuum to afford the crude Propofol with an excellent purity. A subsequent bulb-to-bulb distillation of this residue (140 °C, 5 mbar) is possible to further purify the Propofol, which is obtained as a colorless liquid (58% yield).

^1^H NMR (300 MHz, CDCl_3_) δ 7.07 (dd, *J* = 7.6, 0.5 Hz, 2H), 6.96–6.85 (m, 1H), 4.78 (s, 1H), 3.17 (hept, *J* = 6.8 Hz, 2H), 1.28 (d, *J* = 6.9 Hz, 12H). ^13^C NMR (75 MHz, Chloroform-*d*) δ 149.97, 133.66, 123.48, 120.66, 27.18, 22.78. GC retention time: 13.70 min.

## Figures and Tables

**Table 1:**
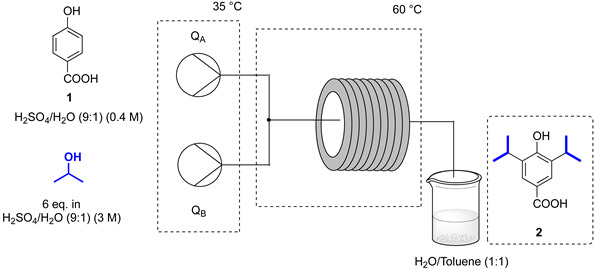
Synthesis of compound **2** in a flow system ^a^.

Entry	Q_A_	Q_B_	Q_T_	Reactor Volume (mL)	*t*^R^ (min)	Yield (%)
	(mL·min^−1^)					
1	0.0625	0.0625	0.125	5	40	70 ^b^
2	0.375	0.375	0.750	30	40	85 ^c^
3	0.750	0.750	1.5	60	40	81 ^c^
4	1.25	1.25	2.5	100	40	84 ^c,d^

^a^ Reactor made with PFA tubing (ID = 1.6mm), AFR^™^ Corning^®^ pump module, T-shaped mixer, elution system H_2_SO_4_/H_2_O (9:1). ^b^ NMR yields using MeNO_2_ as an internal standard. ^c^ Isolated yield. ^d^ 200 mmol/27.6 g scale. Q = flow rate.

**Table 2:**
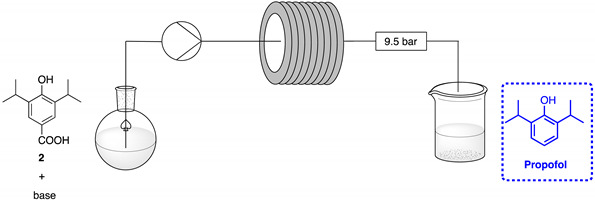
Continuous flow decarboxylation of compound **2** into Propofol ^a^.

Entries	Elution System	Base	Conv. (%) ^a^	Yield (%) ^b^
1 ^c^	Toluene/2-ethoxyethanol (3:1)	TMEDA 1 eq.	>99	60
2 ^c^		TMEDA 2 eq.	>99	61
3 ^c^		DIPEA 1 eq.	>99	13
4 ^c^		DIPEA 2 eq.	>99	17
5 ^c^		TEA 1 eq.	>99	21
6 ^c^		TEA 2 eq.	>99	56
7 ^c^		TEA 6 eq.	>99	59
8 ^d^	Toluene/2-ethoxyethanol (1:1)	TEA 6 eq.	>99	73
9 ^e^	DMF	TEA 6 eq.	>99	77
10 ^f^	2-ethoxyethanol	TEA 6 eq.	>99	86
**11 ^g^**	**2-butoxyethanol**	**TEA 6 eq.**	**>99**	**69**

^a^ Performed in Vapourtec^®^ R4 and R2S device equipped with a copper reactor (ID = 1 mm, Reactor volume = 10 mL, Q = 56 µL·min^−1^, *t*^R^ = 3 h) heated at T = 150 °C. ^b^ NMR yield using MeNO_2_ as an internal standard. ^c^ 0.5 M in toluene/2-ethoxyethanol (3:1). ^d^ 0.5 M in toluene/2-ethoxyethanol (1:1). ^e^ 0.5 M in DMF. ^f^ 0.5 M in 2-ethoxyethanol. ^g^ 0.5 M in 2-butoxyethanol, performed at 200 °C with a flow rate Q = 334 µL·min^−1^ with *t*^R^ = 0.5 h.

## Data Availability

Not applicable.

## Supplementary Materials

The following are available online: ^1^H and ^13^C NMR spectra.

## Sample Availability

Samples of the compounds are not available from the authors.
