# Supplementary material for: Continuous Flow Synthesis of Propofol
Source: Molecules. 2021 Nov 26;26(23):7183. doi: 10.3390/molecules26237183 (PMC8659244; doi:10.3390/molecules26237183)

Continuous Flow Synthesis of Propofol

Romain Mougeot, Philippe Jubault *, Julien Legros *, Thomas Poisson *

Normandie Univ., INSA Rouen

UNIROUEN, CNRS, COBRA (UMR 6014) 76000 Rouen, (France).

SUPPORTING INFORMATION

Table of contents

1. General methods

2. Synthetic procedures

3. Characterizations

1. General methods

All materials were purchased from commercial suppliers. Unless specified otherwise, all reagents and solvents were used as supplied. ^{1}H NMR spectra and ^{13}C NMR spectra were recorded on a Bruker Advance III 300 at 300 MHz and 76 MHz, respectively. Residual solvent peaks were used as the reference. Data for ^{1}H are reported as follows: chemical shift (δ ppm), multiplicity (s = singlet, d = doublet, t = triplet, q = quartet, quint = quintet, sex = sextet, sept = septet, m = multiplet), coupling constant (Hz) and integration). Chemical shifts are reported in ppm relative to the signals corresponding to residual non-deuterated ($CDCl_3$: δ = 7.26 ppm). Analyses were performed on GC-FID thermoscientific trace 1310 equipped with Durabond DB-5MS (30 m, 0.250 mm Ø narrowbore, 0.25 µm film), the He vector gas flow rate of 1ml.min-1 in split mode (50mL.min^{-1}) and the inlet injector temperature of 250 °C. As FID detector, the air flow rate is at 350 mL.min^{-1} with vector gas at 35 mL.min^{-1}. Samples analysis were done, after 2 minutes at 50 °C, at a temperature rate of 10 °C.min^{-1} for 20 minutes reaching 250 °C. All GC-sample were prepared using grade HPLC solvents from Ficher. Distillation were performed on a Büchi Kugelrohr. The flow reactor system is specified for each cases between the AFR™ Corning® module pump device, the Vapourtec® R4 heated system and R2S pump system.

2. Synthetic procedures

a) *Preparation of 3,5-diisopropyl-4-hydroxybenzoic acid (2)*

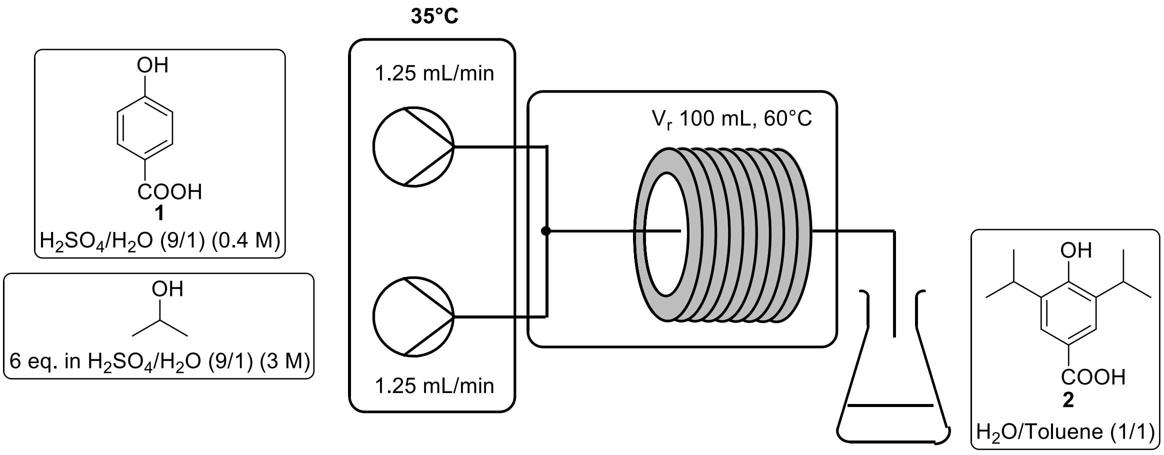

PFA reactor and channels, 1,6mmØ, AFR Corning pump module, T shape mixer, elution system H_2SO_4/H_2O (9/1)

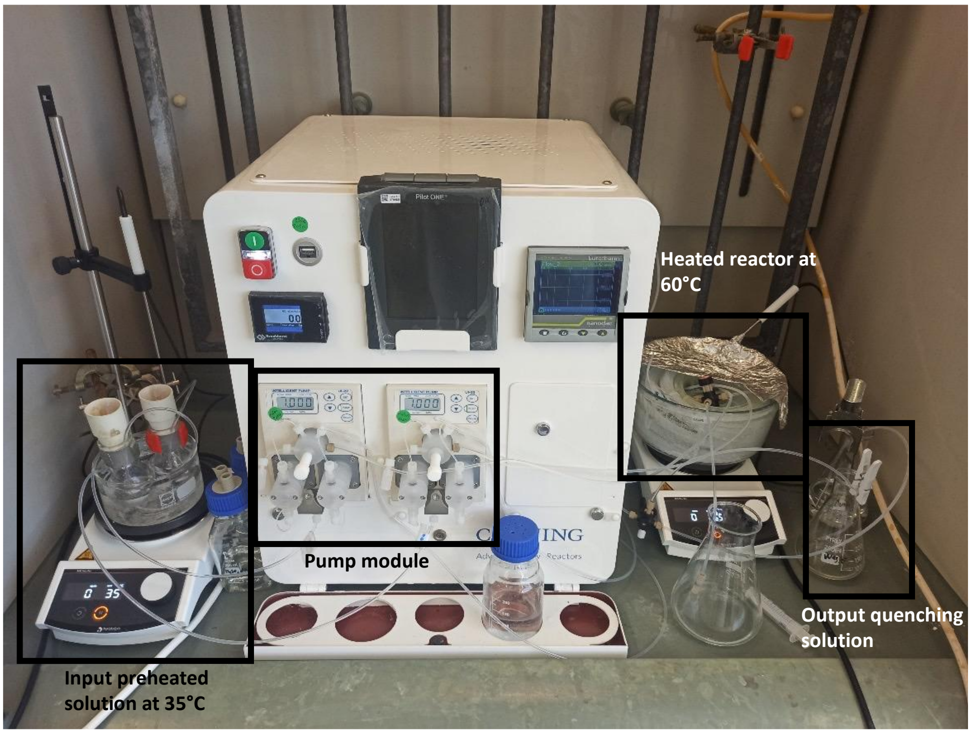

A stock solution of 4-hydroxybenzoic acid (**1**) (200 mmol, 27.6 g) in H_2SO_4/H_2O (9:1, 440 mL/55 mL, 0.5 M) and a stock solution of isopropyl alcohol (6 eq., 1.2 mol, 71.5 g, 91.3 mL) in H_2SO_4/H_2O (9:1, 363 mL/40.7 mL) were heated at 35°C and pumped using the AFR™ Corning® module pump at a flow rate of 1.25 $mL.min^{-1}$ for each solution through a T-mixer heated at 60 °C and a 100 mL coil PFA reactor also heated at 60 °C. The output of the reactor was collected into a stirred solution of H_2O/toluene (1:1) at room temperature. The setup was eluted by a solution of H_2SO_4/H_2O (9:1). The layers were separated and the aqueous phase was extracted with toluene. The combined organics phases were washed with brine, dried with $MgSO_4$ and concentrated under vacuum. Flash chromatography afforded the desired product **2** as a white solid (84%, 168 mmol, 37.3 g).

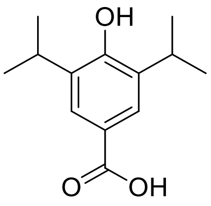

2

3,5-diisopropyl-4-hydroxybenzoic acid (2): ^{1}H NMR (300 MHz, Chloroform-*d*) δ 7.87 (s, 2H), 3.17 (hept, *J* = 6.9 Hz, 2H), 1.31 (d, *J* = 6.9 Hz, 12H). ^{13}C NMR (75 MHz, Chloroform-*d*) δ 172.64, 155.15, 133.73, 126.50, 121.47, 27.22, 22.60. Melting point: 139-142°C. GC retention time: 19.02 minutes. Data were in accordance with those in literature.[1]

[1] Pramanik, C.; Kotharkar, S.; Patil, P.; Gotrane, D.; More, Y.; Borhade, A.; Chaugule, B.; Khaladkar, T.; Neelakandan, K.; Chaudhari, A.; Kulkarni, M. G.; Tripathy, N. K.; Gurjar, M. K. *Org. Process Res. Dev.* **2014**, *18*, 152–156.

b) Preparation of Propofol

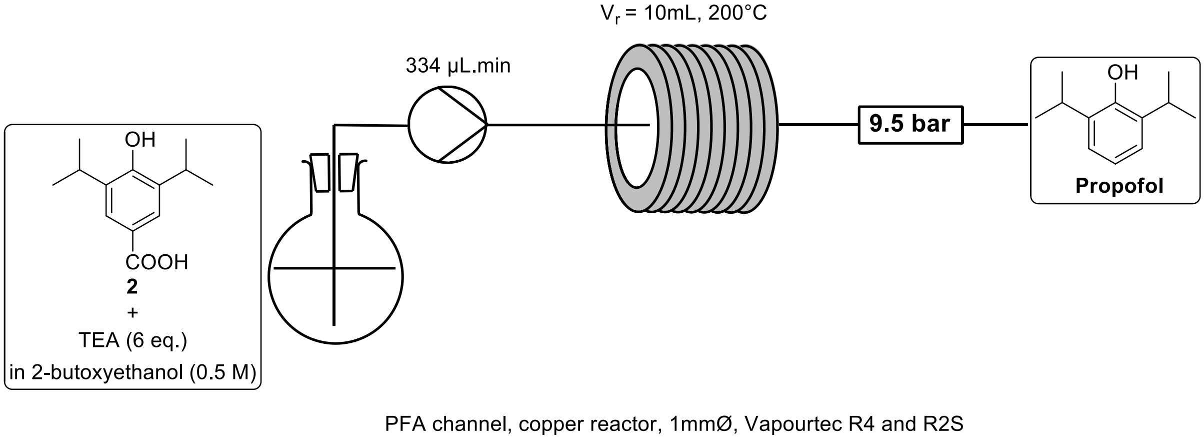

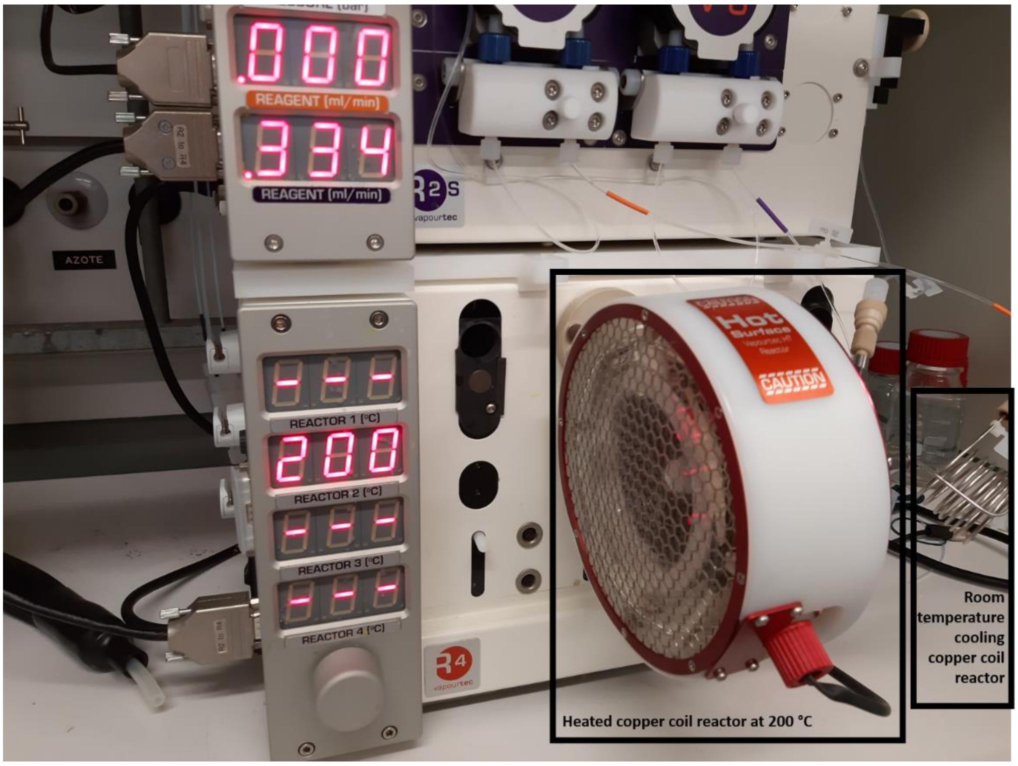

To a stock solution of 3,5-diisopropyl-4-hydroxybenzoic acid (**2**) (0.5 mmol, 111 mg, 1 eq.) in 2-butoxyethanol (500 µL) was added triethylamine (3 mmol, 500 µL, 6eq.). The mixture was stirred at room temperature and pumped by R2S Vapourtec® pump device at a flow rate of 334 $\mu L.min^{-1}$ through a 10 mL copper coil reactor at 200 °C and a 1 mL copper coil reactor at room temperature. The setup was eluted by 2-butoxyethanol. The output of the reactor was collected into a stirred solution of brine/cyclohexane. The organic layer was dried with $MgSO_4$ and concentrated under vacuum to afford the crude Propofol with an excellent purity. A subsequent bulb to bulb distillation of this residue (140°C, 5 mbar) is possible to further purify the Propofol, which is obtained as a colourless liquid. (58% yield)

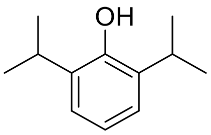

Propofol

Propofol: ^{1}H NMR (300 MHz, Chloroform-*d*) δ 7.07 (dd, *J* = 7.6, 0.5 Hz, 2H), 6.96 – 6.85 (m, 1H), 4.78 (s, 1H), 3.17 (hept, *J* = 6.8 Hz, 2H), 1.28 (d, *J* = 6.9 Hz, 12H). ^{13}C NMR (75 MHz, Chloroform-*d*) δ 149.97, 133.66, 123.48, 120.66, 27.18, 22.78. GC retention time: 13.70 minutes. Data were in accordance with those in literature.[1]

3. Characterizations

^{1}H NMR spectra of **2**

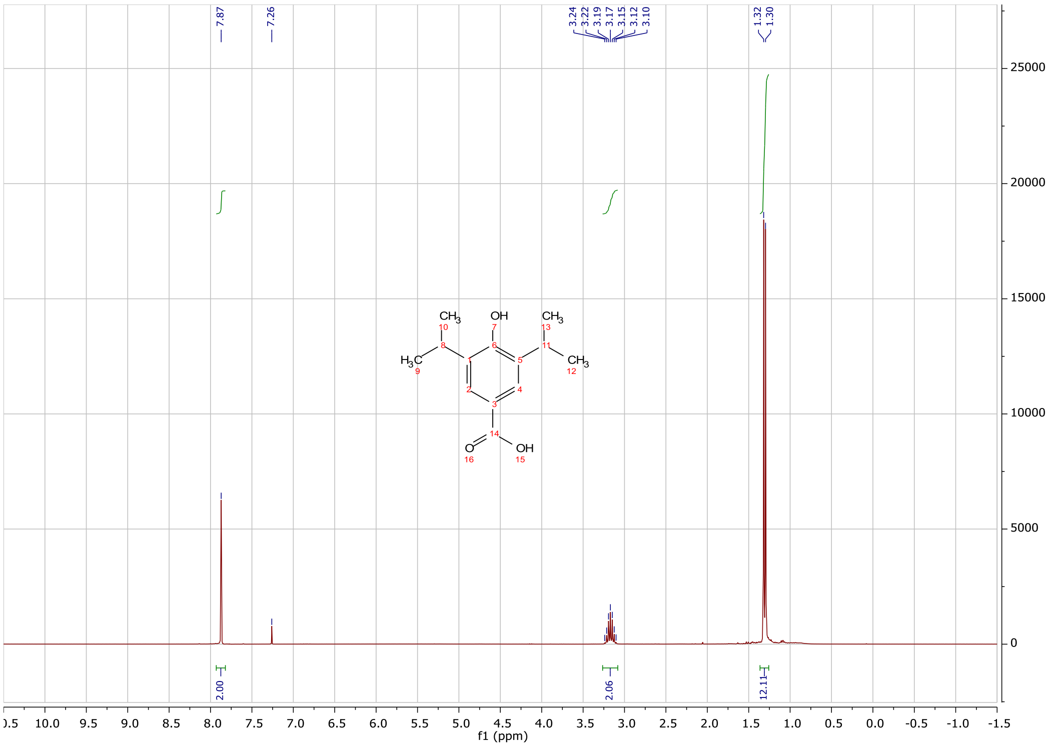

^{13}C NMR spectra of **2**

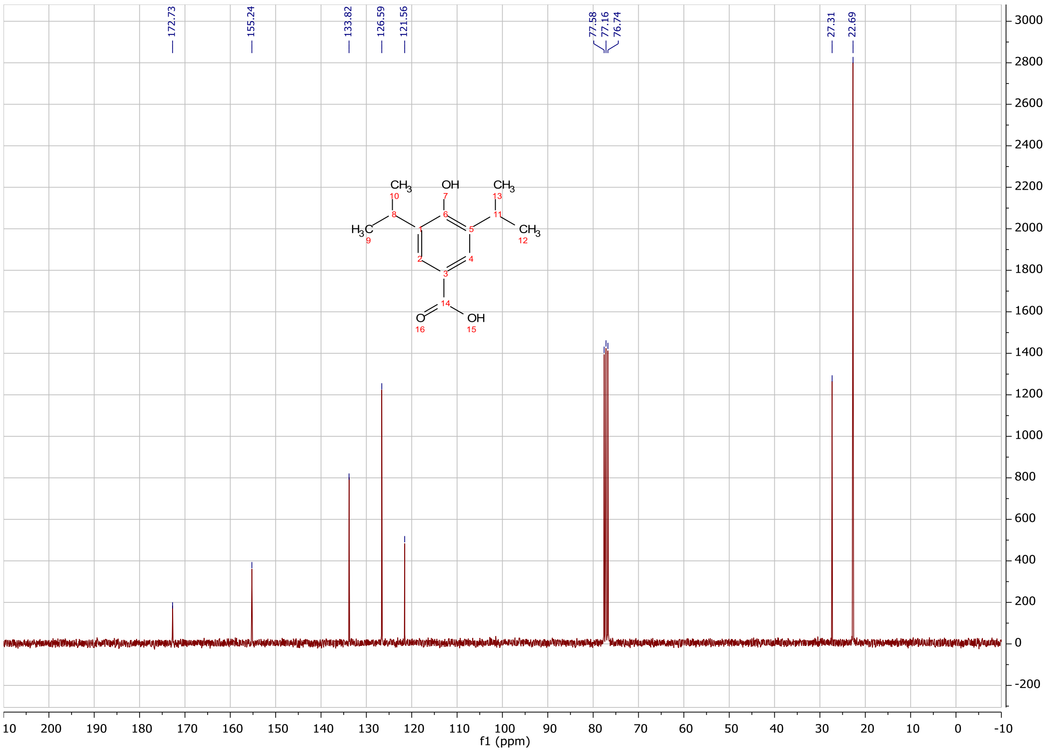

GC chromatograph of **2**

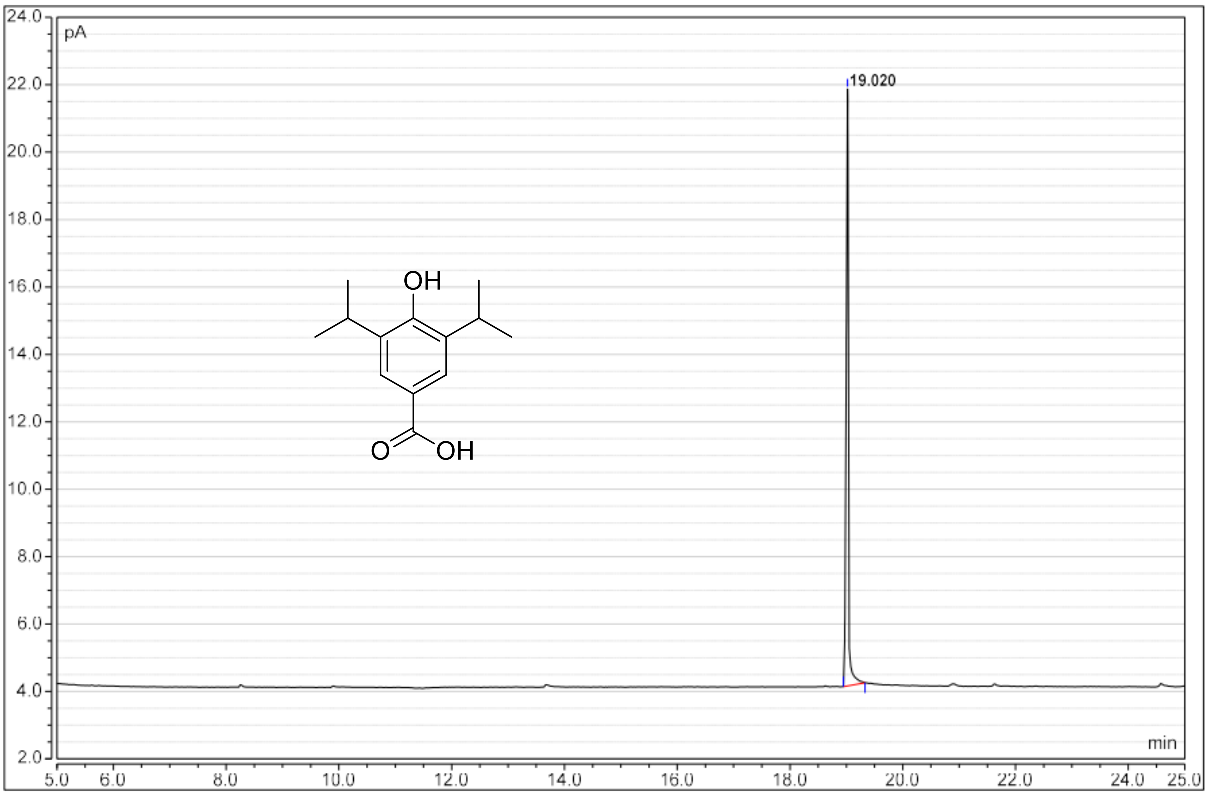

^{1}H NMR spectra of **propofol**

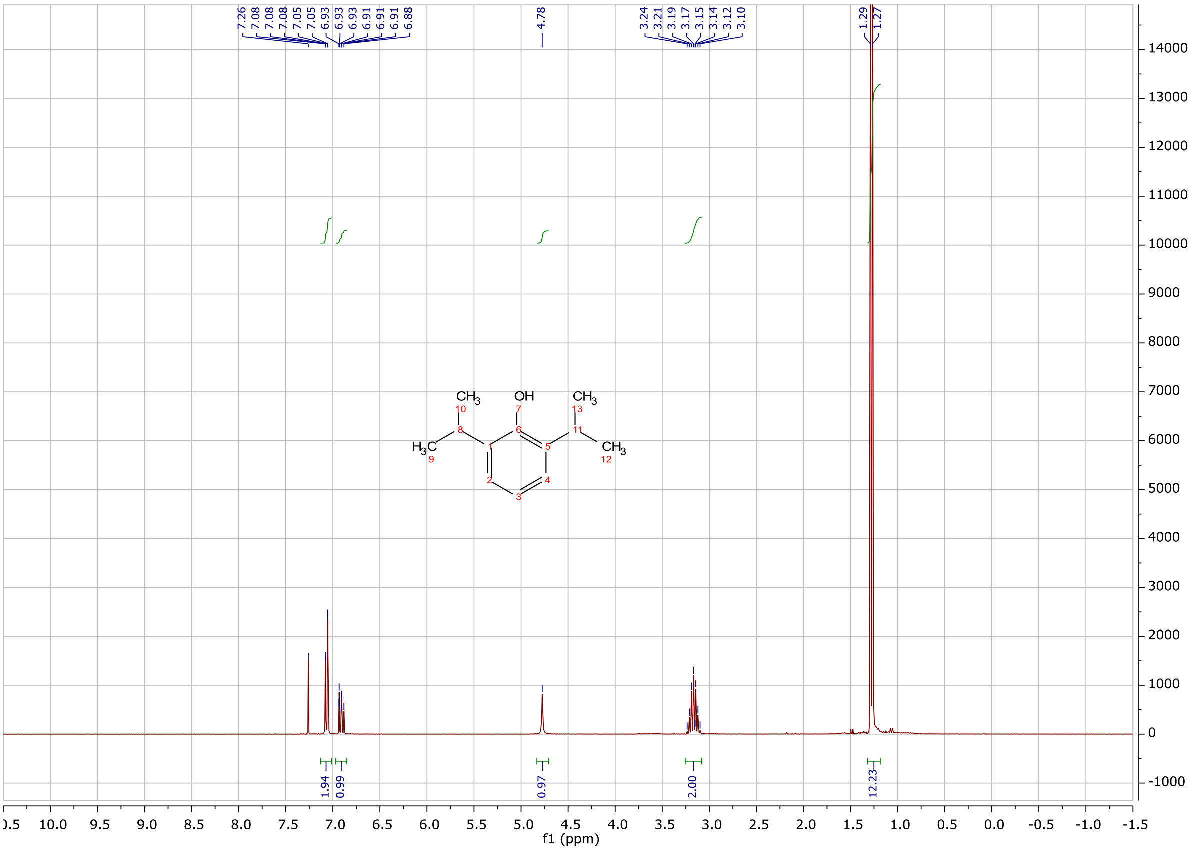

^{13}C NMR spectra of **propofol**

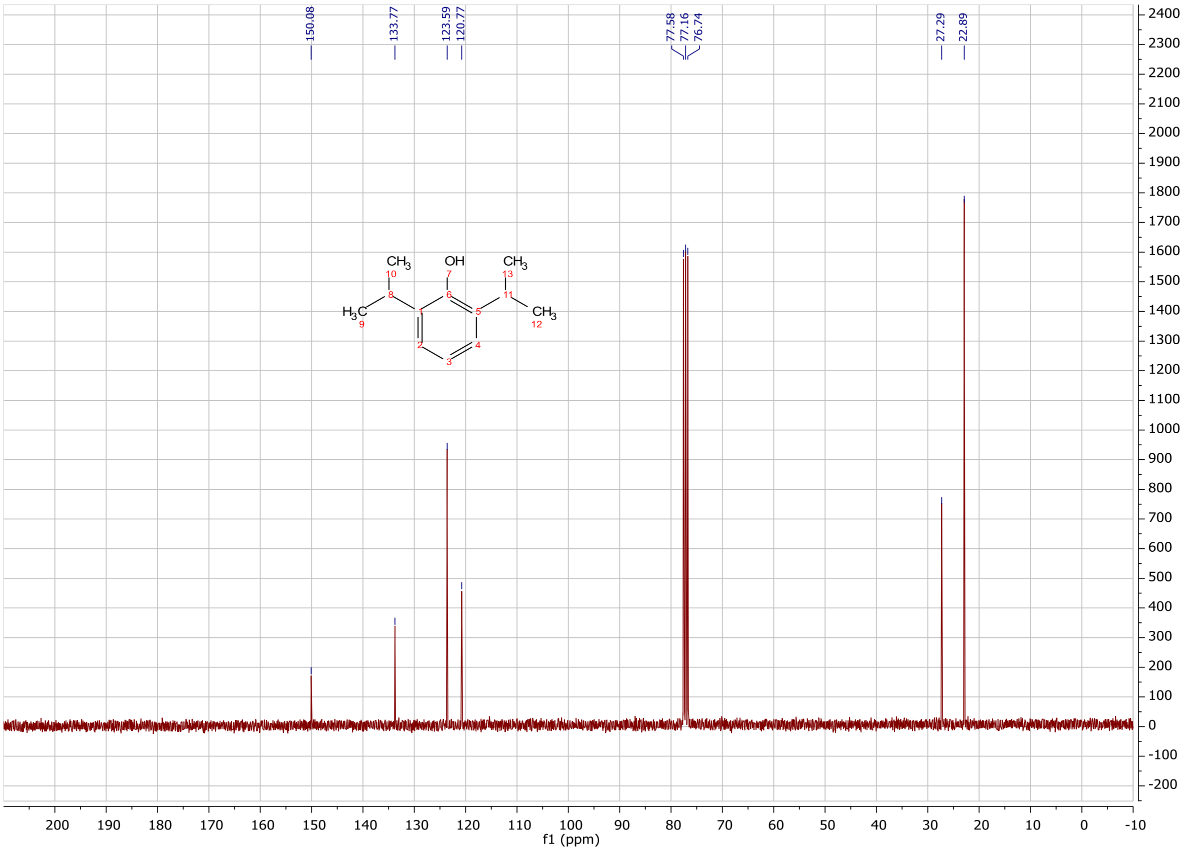

GC chromatograph of **propofol**

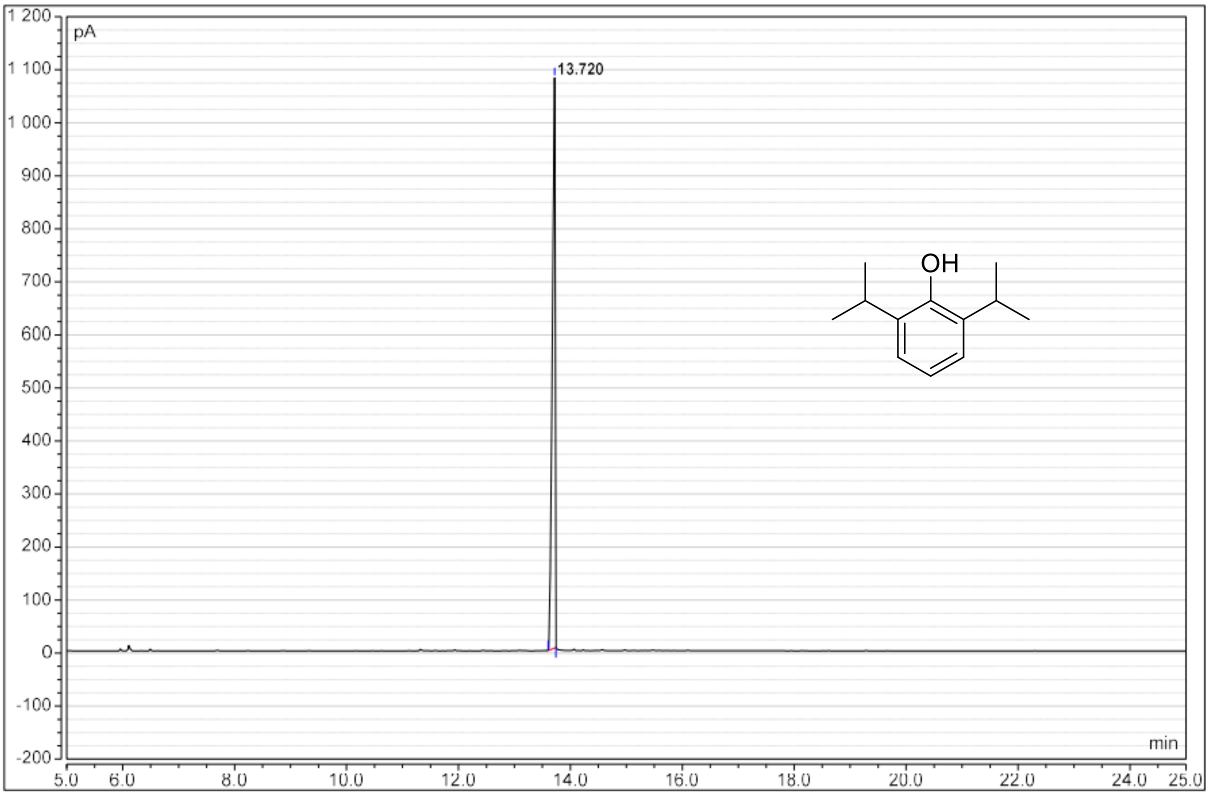

GC chromatogram of crude mixture of **propofol**

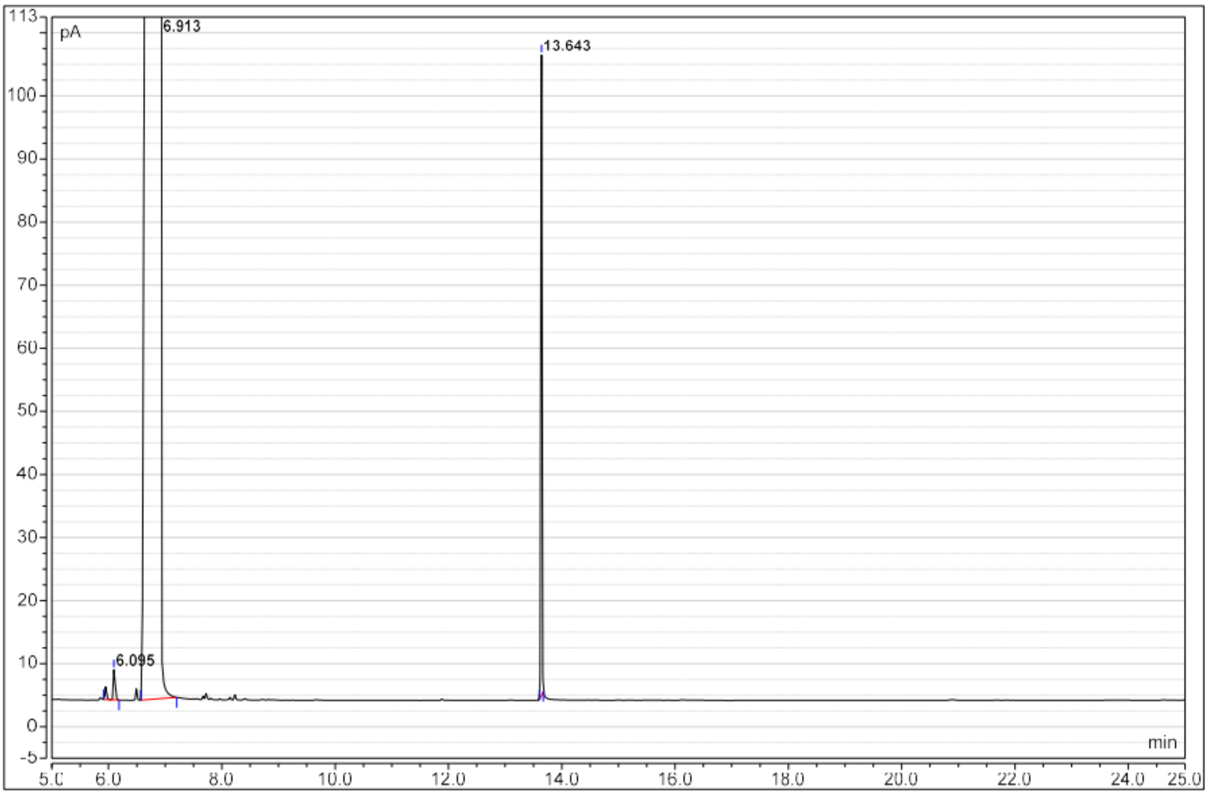

Supplement: Supplementary file 1 [file molecules-26-07183-s001.zip › molecules-1470690-supplementary.pdf]
